# The evidence is in: accountability needs to be injected into the policy-making process for household food insecurity reduction

**DOI:** 10.24095/hpcdp.45.9.04

**Published:** 2025-09

**Authors:** Valerie Tarasuk, Lynn McIntyre

**Affiliations:** 1 Department of Nutritional Sciences, Temerty Faculty of Medicine, University of Toronto, Toronto, Ontario, Canada*; 2 Department of Community Health Sciences, Cumming School of Medicine, University of Calgary, Calgary, Alberta, Canada*

**Keywords:** food insecurity, Canada, public policy, food assistance

## Abstract

As the problem of household food insecurity perseveres, effective evidence-informed responses are badly needed. The systematic reviews of evidence compiled by the Public Health Agency of Canada provide an important foundation for such action, but they also indicate the need for accountability, so that precious public funds do not continue to be spent on initiatives with no evidence of impact. We need targets for food insecurity reduction and some accountability for policy interventions that come with significant public investments. Household food insecurity rates and the related adverse consequences are only going to get worse unless we address the inadequate, insecure incomes that are the primary driver of this population health problem.

**Highlights**

- Household food insecurity is growing in Canada and effective evidence-informed responses to this problem are badly needed.
- Accountability in policy making is essential to ensure that scarce public funds are not allocated to initiatives that lack evidence of impact.
- Many policy interventions that increase the incomes of low-income households have been shown to reduce household food insecurity; there is no such evidence base for food-based interventions.
- To increase accountability and incentivize effective, evidence-informed income interventions that address food insecurity, we propose that the federal government commit to reducing food insecurity by 50% and eliminating severe food insecurity by 2030.

## Introduction

**Figure 1:**
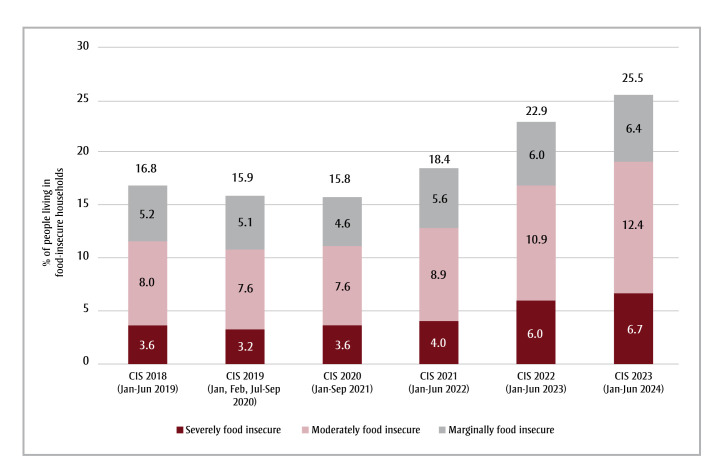
Percentage of people living in food-insecure households in Canada, excluding the territories, 2019–2024

Household food insecurity affected 25.5% of people in the Canadian provinces and 37.4% of people in the territories in 2024.^1^ The recent rise may reflect inflationary pressures, but even during the low inflation period before the COVID-19 pandemic, food insecurity was widespread (Figure 1).^2^ Briefly defined as the lack of access to adequate food because of financial constraint, food insecurity is most prevalent among households with low incomes, renters, social assistance recipients, lone-parent female-led families and those who identify as Black or Indigenous.^3^

**Data source:** Statistics Canada, Table 13-10-0834-01: Food insecurity by economic family type.^7^

**Abbreviation:** CIS, Canadian Income Survey. 

**Note:** The CIS year refers to the 12-month period prior to when the CIS interview took place. The survey collection periods are shown in parentheses.

Food insecurity is a serious population health problem in Canada, associated with poorer mental health, increased risk of infectious and noncommunicable diseases and injuries, poorer disease management, higher health care use and premature mortality.^4^ The persistently high and now escalating prevalence of household food insecurity suggests an urgent need for effective, evidence-informed policy interventions.

Against this backdrop, staff at the Public Health Agency of Canada (PHAC) Centre for Surveillance and Applied Research led an exhaustive review of evidence to identify effective intervention strategies to reduce household food insecurity in Canada. The authors of this commentary participated on a voluntary basis as subject matter experts, an experience that was positive and informative as we observed unfailing sophistication in the review methods deployed. Yet, as is appropriate for a government science-led process, these reviews end without critical interpretation or recommendations. In this commentary, we discuss what we see as the most important policy and research implications stemming from PHAC’s findings. We do so in the belief that PHAC’s evidence review of interventions should not be ignored and that household food insecurity is not an intractable problem; it persists because its reduction is not an explicit policy goal of governments.

PHAC’s evidence reviews comprise five peer-reviewed manuscripts,^5^-^9^ ranging from the impact of COVID-19 on food insecurity^6^ to a comprehensive review of Nutrition North Canada, a federal retail subsidy program intended to support food affordability in northern Canada.^7^ Taken together, these reviews offer clear direction on what works and what does not work to reduce food insecurity in Canada. But they also raise some troubling questions about current government responses. 

Here we comment on the findings from the reviews of public policy interventions in the general population^1^ and food-based interventions^9^ because we see these evidence reviews as most germane to population health outcomes. We synthesize the main findings, identify priorities for policy intervention and suggest directions for future research.

## Main findings

***The effectiveness of income supplementation***

The most important finding to emerge from this extensive review of the evidence is the effectiveness of federal and provincial policy interventions that modestly increase the incomes of Canadians living at low income, for example, Old Age Security pensions, the Canada Child Benefit (CCB) and social assistance. Idzerda et al. concluded with moderate to high certainty that income supplementation reduces food insecurity, but they found no evidence that the assessed housing assistance programs and food retail subsidy programs had any impact.^5^ Their findings suggest that inadequate and insecure incomes are the primary driver of household food insecurity and that income support policies are key to reducing this population health problem.^5^

As indicated by the moderate to high certainty rating, the quality of the research on income supplementation is robust.^5^ Researchers have been able to estimate changes in the probability of food insecurity among population subgroups exposed to specific income interventions, often employing complex econometric methods to account for other well-established influences on households’ food insecurity status (e.g. housing tenure, household composition, education, province or territory of residence). The strength of the evidence garnered from these studies highlights the fallacy in discounting the importance of income as a solution to Canada’s food insecurity problem simply based on recent reports that most food-insecure households have incomes above the official poverty line.^10^,^11^

In their systematic review, Idzerda et al. found that housing assistance (primarily rent subsidies) had no effect on food insecurity.^5^ Since 2020, when the last of the studies reported findings, a variety of housing interventions have been proposed by multiple levels of government in response to the housing affordability crisis. The impact of new affordable housing interventions should be ready to be evaluated for their effects on household food insecurity.

***Food-based interventions fail to reduce food insecurity***

Food banks have dominated Canada’s response to food insecurity for more than 40 years and received unprecedented federal and provincial funding throughout the COVID-19 pandemic.^12^ Several jurisdictions, for example, British Columbia, Alberta, Quebec and New Brunswick, continue to allocate significant funds to these programs. Yet there has been almost no evaluation of the effectiveness of charitable food assistance programs in reducing food insecurity. The few studies that do exist are of low quality and provide no systematic review evidence of effectiveness.^9^ A parallel body of literature has charted the very low rates of utilization of food charity programs by domiciled, food-insecure households. This is unsurprising given that charitable food assistance does not alter the underlying drivers of households’ food insecurity. The conclusions of Idzerda et al.’s systematic review^9^ are consistent with what many leaders in this sector have been saying for years—that the solutions to the desperation that drives people to seek food bank assistance lie in public policy reforms that address the underlying income issues.^13^-^15^

Similar to the literature on food charities, the existing evaluations of alternative food programs including food box, gardening and school food programs and hunting and fishing interventions specific to Indigenous populations, while limited in number, yielded no evidence of effectiveness.^9^ The two available evaluations of food voucher programs provided low-to-moderate evidence that these programs may reduce food insecurity, but this could be explained by the fact that the vouchers increase the purchasing power of participating households, albeit on a small, and often time-limited, scale. Political claims that the recent one-billion-dollar investment in a National School Food Program will reduce the number of “hungry children” will need to be assessed over the next few years against food insecurity rates in households with children.^16^ The lack of evidence that food programs are effective in reducing food insecurity should also be a cautionary tale for groups seeking food sovereignty for their communities. These groups perhaps should not expect different results.

We have 40 years of food-based initiatives for people experiencing food insecurity, but fewer evaluative studies than we have provinces and territories in Canada. While government science itself shows that these policy directions are not evidence-based, we will inevitably see further investments in food-based interventions as media and political eyes focus on rising rates of household food insecurity. As social scientists continue to unpack why these ideas keep on drawing resources, inhibiting more effective solutions,^15^,^17^-^19^ we need to ask why no evidence is needed and what are the intended outcomes of food-based initiatives if not a reduction in household food insecurity? Indeed, Agriculture and Agri-Food Canada’s 2024 evaluation of their Local Food Infrastructure Fund suggests that the outcome of resource support for the food insecure–serving sector may not be the reduction of food insecurity but rather alleviation of organizational stress brought on by increased client demand for their services.^17^

## Implications for future policy responses

This is a time for reckoning. There is little to no evidence of food-based interventions having an impact on household food insecurity.^9^ Yet the fund-raising communications of charitable and alternative food providers explicitly claim that they are addressing or preventing food insecurity (see, for example^18^-^20^). Scholars have also commented on underlying corporatization as justifying food-based interventions.^21^,^22^ Interdisciplinary research could reveal more about these dynamics, but surely the first step is to call out the incongruity of food-based interventions as a strategy for food insecurity reduction now that the absence of evidence has been revealed. We believe that the related lack of accountability for the impact of recent investments in food charity (e.g. pandemic-related federal programs^23^ and the ongoing Local Food Infrastructure Fund^17^) is part of the explanation for the persistently high prevalence of food insecurity in Canada. No government has seriously tried to reduce food insecurity.

The positive effects of income-based interventions on household food insecurity documented by Idzerda et al.^5^ derive from assessments of federal and provincial programs that were not explicitly designed to prevent or reduce food insecurity. The effects found were incidental to other policy goals. The results of the systematic review consequently do not tell us what the optimal design of an income intervention that minimizes household food insecurity might be nor the extent to which income supplementation alone could reduce food insecurity prevalence. And, as with any public policy intervention, additional equity considerations need to be evaluated—who benefits, who is missed, whether disparities increase or decrease, and whether targeted approaches need to augment universalist implementation?^24^

It is time for the reduction of the prevalence and severity of household food insecurity to become a deliberate policy goal in Canada. We propose that the federal government commit to the elimination of severe food insecurity in Canada and a 50% reduction in the 2024 prevalence of household food insecurity by 2030. The elimination of severe food insecurity is effectively Sustainable Development Goal 2: Zero Hunger, to which Canada has committed.^25^ This Sustainable Development Goal matters because (1) severe food insecurity is extremely damaging to health (e.g. adults in Canada who experience severe food insecurity die on average 9years earlier than those who are food secure^26^); (2) the prevalence of severe food insecurity is rising; and (3) making the elimination of food insecurity a discrete policy goal is essential to ensure that the implemented interventions actually reach and help those affected.^27^ The elimination of severe food insecurity is a realistic goal given its high sensitivity to income interventions.^28^,^29^

Accountability needs to be injected into the policy-making process. With the insertion of food insecurity measurement into the Canadian Income Survey and its inclusion on the Poverty Dashboard of Indicators, the necessary tracking indicators are already in place to evaluate income intervention policy against food insecurity prevalence and severity outcomes. Through an iterative process of intervention and evaluation, using longitudinal studies with repeat measures, the crucial policy levers to minimize household food insecurity in Canada can be honed. The federal department best placed to lead this work is Employment and Social Development Canada.

Reducing food insecurity and eliminating severe food insecurity require both federal and provincial or territorial policy reforms because both levels of government are responsible for income transfers that, depending on their design, can support or compromise household food security. Old Age Security pensions and the Guaranteed Income Supplement provide vital protection against food insecurity for older adults who rely on these programs.^27^ But with food insecurity rates beginning to creep up among older adults,^1^ it is imperative to maintain the protective effect of public pensions. In addition, the principles of income adequacy and security that define the Old Age Security/Guaranteed Income Supplement need to be applied to federal and provincial or territorial income support programs for working-age adults and their children. This means ensuring that the CCB enables families living at lower income to afford the food they need. With 32.9% of children aged younger than 18 years now living in families experiencing food insecurity,^1^ the CCB urgently needs review. Reviews of Employment Insurance and the Canada Workers Benefit are also required to ensure that these benefits are sufficient to enable recipients to maintain household food security despite involuntary unemployment or underemployment. Provincial and territorial policies relevant to food insecurity include minimum wage, taxation, child and family benefits, and social assistance.^28^,^30^,^31^ A Basic Income Guarantee^32^ could replace many of these federal and provincial and territorial programs and should certainly be evaluated against food insecurity outcomes.

## Conclusion

As the problem of household food insecurity continues to grow, effective evidence-informed responses are badly needed. The systematic reviews of evidence compiled by PHAC provide an important foundation for such action. But the results of these evidence reviews also lay bare the need for accountability, so that no more public funds are wasted on initiatives with no evidence of impact under the guise of addressing food insecurity. We need targets for food insecurity reduction and some accountability for policy interventions that come with significant public investments. Household food insecurity rates and the related adverse consequences are only going to get worse unless we address the inadequate and insecure incomes that are the primary driver of this population health problem.

## Acknowledgements

The authors are indebted to Tim Li for his assistance with the preparation of Figure 1. His work is supported by project grant funding from the Canadian Institutes of Health Research PJT-178380.

## Conflicts of interest

The authors declare no conflicts of interest.

## Authors’ contributions and statement

VT: Conceptualization, writing—original draft, writing—review and editing.

LM: Conceptualization, writing—original draft, writing—review and editing.

The content and views expressed in this article are those of the authors and do not necessarily reflect those of the Government of Canada.
